# Latent TGF-β1 protects against diabetic kidney disease via Arkadia/Smad7 signaling

**DOI:** 10.7150/ijbs.61647

**Published:** 2021-08-19

**Authors:** Weifeng Wu, Xiao R. Huang, Yongke You, Liang Xue, Xiao-Jing Wang, Xiaoming Meng, Xiang Lin, Jiangang Shen, Xueqing Yu, Hui-Yao Lan, Haiyong Chen

**Affiliations:** 1 School of Chinese Medicine, Li Ka Shing Faculty of Medicine, The University of Hong Kong, Hong Kong, China.; 2 Department of Medicine and Therapeutics, Li Ka Shing Institute of Health Sciences, The Chinese University of Hong Kong, Hong Kong, China.; 3 Guangdong-Hong Kong Joint Laboratory on Immunological and Genetic Kidney Diseases, Guangdong Academy of Medical Sciences, Guangdong Provincial People's Hospital, Guangzhou, China.; 4 Department of Pathology, University of Colorado Denver, Aurora, CO, United States.; 5 School of Pharmacy, Anhui Medical University, Anhui, China.

**Keywords:** Latent TGF-β1, inflammation, fibrosis, Arkadia, Smad7, Diabetic kidney disease

## Abstract

TGF-β1 has long been considered as a key mediator in diabetic kidney disease (DKD) but anti-TGF-β1 treatment fails clinically, suggesting a diverse role for TGF-β1 in DKD. In the present study, we examined a novel hypothesis that latent TGF-β1 may be protective in DKD mice overexpressing human latent TGF-β1. Streptozotocin-induced Type 1 diabetes was induced in latent TGF-β1 transgenic (Tg) and wild-type (WT) mice. Surprisingly, compared to WT diabetic mice, mice overexpressing latent TGF-β1 were protected from the development of DKD as demonstrated by lowing microalbuminuria and inhibiting renal fibrosis and inflammation, although blood glucose levels were not altered. Mechanistically, the renal protective effects of latent TGF-β1 on DKD were associated with inactivation of both TGF-β/Smad and nuclear factor-κB (NF-κB) signaling pathways. These protective effects were associated with the prevention of renal Smad7 from the Arkadia-induced ubiquitin proteasomal degradation in the diabetic kidney, suggesting protection of renal Smad7 from Arkadia-mediated degradation may be a key mechanism through which latent TGF-β1 inhibits DKD. This was further confirmed *in vitro* in mesangial cells that knockdown of Arkadia failed but overexpression of Arkadia reversed the protective effects of latent TGF-β1 on high glucose-treated mesangial cells. Latent TGF-β1 may protect kidneys from TGF-β1/Smad3-mediated renal fibrosis and NF-κB-driven renal inflammation in diabetes through inhibiting Arkadia-mediated Smad7 ubiquitin degradation.

## Introduction

Diabetic kidney disease (DKD) is the most common diabetic complication and a major cause of end-stage renal disease (ESRD) worldwide. DKD accounts for almost 50% of the new cases of ESRD in developed countries 1. TGF-β1 has long been considered as a key mediator in DKD 2. It has been shown that anti-TGF-β1 treatment with neutralizing antibodies can inhibit DKD experimentally 3,4. Disappointingly, a recent clinical trial study using a humanized monoclonal neutralizing antibody against TGF-β1 (LY2382770) has been proven no efficacy on patients with DKD 5. These discrepancies between experimental and clinical outcomes suggest a diverse role for TGF-β1 in DKD, although mechanisms are largely unclear.

It is well known that TGF-β1 contains latency and active forms. Latent TGF-β1 consists of a C-terminal dimer of mature TGF-β1, and an N-terminal dimer known as latency-associated peptide (LAP) 6,7. The active TGF-β1 is released when the N-terminal LAP is cleaved from the latent TGF-β1 6,8. In the context of fibrosis, active TGF-β1 is pathogenic as mice overexpressing an active form of TGF‐β1 in the liver develop progressive liver and renal fibrosis 2,9. In contrast, latent TGF-β1 is protective as mice overexpressing latent TGF-β1 are protected against obstructive kidney disease 10, crescentic glomerulonephritis 11, and bleomycin-induced lung injury 12. All these studies suggest an opposite role for active versus latent TGF-β1 in the pathogenesis of kidney diseases. Thus, we hypothesized that latent TGF-β1 may be protective in DKD. This was tested in the study in a mouse model of type 1 diabetes induced by STZ in mice overexpressing the human latent TGF-β1. The role of latent TGF-β1 and potential mechanisms in DKD were investigated *in vivo* and *in vitro*.

## Materials and methods

### Animals

All animal protocols and procedures were approved by the Committee on the Use of Live Animals in Teaching and Research (CULATR) at the University of Hong Kong. The latent TGF-β1 transgenic (Tg) mice were generated in ICR background mice by functionally inserting the human TGF-β1 gene using K5 promoter plasmid as previously described 13. The male latent TGF-β1 Tg and littermate wild-type (WT) male mice, aged 10 - 12 weeks, were randomly divided into normal and diabetes mellitus (DM) groups (n=6). All the mice were maintained in standard animal housing with a 12-h light/dark cycle and were euthanized by intraperitoneal injection of ketamine/xylene (80 mg/kg and 10 mg/kg respectively) after the experiments.

### STZ-induced type 1 diabetes

Mice received a daily intraperitoneal injection of 50 mg/kg STZ for 5 consecutive days. STZ was freshly prepared in 0.1 M sodium citrate buffer (pH 4.5) at a concentration of 7.5 mg/ml. The low-dose STZ induction protocol was employed as recommended by the Animal Models of Diabetic Complications Consortium (https://www.diacomp.org/). Kidneys and blood were harvested at 16 weeks after diabetes induction.

### Urinary albumin excretion

For urine analysis, 24-h urine samples were collected from metabolic cages every 2-4 weeks. Urinary albumin was measured using a competitive ELISA kit according to the manufacturer's instructions (Exocell, Philadelphia, PA, USA) and urinary creatinine was measured by the Creatinine Companion kit (Exocell, Philadelphia, PA, USA) according to the manufacturer's instructions. Urinary albumin excretion (UAE) was measured based on the total urinary albumin/creatinine ratio.

### Histology and immunohistochemistry

Histology and immunohistochemistry analyses were performed using 4% paraformaldehyde (PFA)'s-fixed, paraffin-embedded tissue sections (5 μm) as previously described 14-16. Mesangial matrix expansion was measured by periodic acid Schiff (PAS) staining. The renal tubulointerstitial injury was scored by the percentage of injured area with tubular dilation, interstitial expansion and tubular atrophy: 0 = normal; 1 = 1-10%; 2 = 10-25%; 3 = 26-50%; 4 = 51-75%; 5 = 75-95%; 6 = >96%. The extracellular matrix deposition (ECM) was examined by ten random view fields (at 400×) for each kidney section stained with Masson's trichrome. The ratio of ECM area to total field area was analyzed by ImagePro Plus 7.0 (Media Cybernetics, Rockville, MD, USA). Tissue sections were incubated with primary antibodies targeting F4/80 (AbD Serotec; catalog # MCA497; dilution 1:500), phospho-Smad2/Smad3 (Santa Cruz; catalog # sc-11769; dilution 1:500), IL-1β (Abcam; catalog # ab9722; dilution 1:100), Fibronectin (Abcam; catalog # ab2413; dilution 1:100), Collagen I (Abcam; catalog # ab34710; dilution 1:100), Collagen IV (Abcam; catalog # ab6586; dilution 1:100) or phospho-p65 (Cell Signaling Technology; catalog # 3037; dilution 1:500) overnight at 4 °C, and then incubated with horseradish peroxidase (HRP)-labeled anti-rat or anti-rabbit secondary antibody. Next, diaminobenzidine was added, and the positive cell areas (stained brown) were quantitatively analyzed with ImagePro Plus 7.0 (Media Cybernetics, Rockville, MD, USA) as previously described 14. In brief, the positive area on stained kidney tissue sections was quantitatively calculated as the percentage of positively stained area to the total field area examined in at least 8 fields of views for each section. Positive cells of F4/80, phospho-Smad2/3 and phospho-NF-κB/p65 were enumerated in 20 consecutive glomeruli and expressed as cells/glomerular cross-section (gcs), whereas positive cells in the tubulointerstitium were counted under high-power fields (400×) using a 0.0625-mm^2^ graticule fitted in the eyepiece of the microscope and expressed as the number of positive cells/mm^2^ by quantifying 10 to 20 fields of views for each section.

### *In vitro* experiments

Mouse mesangial cells (MCs) and tubular epithelial cells (mTECs) were from Prof. Hui-Yao Lan's lab and cultured in Dulbecco's modified Eagle's medium (DMEM)/Ham's F12 medium (Invitrogen Life Technologies, Carlsbad, CA, USA) containing 10% FBS at 37 °C under an atmosphere of 5% CO_2_. Cells were seeded in six-well plates at a density of 2×10^5^ cells/well, and then cultured in FBS-free medium for 24 h and stimulated with recombinant human latent TGF-β1 protein (20 ng/ml; R&D Systems, Minneapolis, MN, USA) or latency-associated peptide (15 ng/ml, R&D Systems, Minneapolis, MN, USA) under low (5.5 mM) or high (35 mM) D-glucose conditions for up to 6 h; D-mannitol (29.5 mM) was used as an osmotic control. The cells were harvested to detect the levels of fibronectin, Collagen I, Collagen IV, IL-1β, phospho-NF-κB/p65, phospho-Smad3, Arkadia, and Smad7 protein, as well as the mRNA expression of fibronectin, TNF-α, and Arkadia. All experiments were repeated independently at least three times.

### Cell transfection

The knockdown or overexpression of Arkadia was confirmed by western blot analysis after transfecting MCs with mouse-derived Arkadia siRNA (Integrated DNA Technologies, Inc., Coralville, IA, USA) or with an overexpression plasmid (GFP-Arkadia/RNF111; Addgene plasmid #112228; http://n2t.net/addgene:112228; RRID: Addgene_112228), respectively. Briefly, cells were seeded in six-well plates and transfected with Arkadia siRNA/plasmid or a negative control mixed with Lipofectamine 3000 transfection reagent (Invitrogen, Carlsbad, CA, USA) for up to 24 h according to the manufacturer's instructions. The cells with knockdown or overexpression of Arkadia were then treated with recombinant human latent TGF-β1 protein (20 ng/mL; R&D Systems) under low (5.5 mM) or high (35 mM) D-glucose conditions for up to 6 h, and subsequently harvested for western blot analysis.

### RNA extraction and RT-PCR

Total RNA was extracted from kidneys or harvested cells using an RNA extraction kit (RNeasy; Qiagen, Valencia, CA, USA). Real-time PCR was performed in a total volume of 9 µL, which included 2 µL cDNA solution, 4 µL Bio-Rad iQ SYBR Green supermix with Opticon 2 (Bio-Rad, Hercules, CA, USA), 2.4 µL nuclease-free water, and 0.6 µL of each primer. The mRNA levels were determined by RT-PCR as previously described 14,15, using the following thermal cycling conditions: 95 °C for 2 min, 40 cycles of 95 °C for 5 s, 58 °C for 20 s, and 72 °C for 20 s. The mRNA levels of collagen IV (Col IV), fibronectin (FN), TNF-α, IL-1β, and Arkadia were analyzed. The relative levels of gene expression were calculated by a ∆∆CT method and normalized to the housekeeping gene, β-actin. The ratio of the mRNA was expressed as mean ± SEM. The primers used in this study are shown in **Supplementary Table S1**.

### Western blot analysis

Protein extraction from kidneys or cells using RIPA buffer and western blot analysis were performed as previously described 14,15. Primary antibodies used in this study included those against phospho-Smad3, Smad3 (Cell Signaling Technology, MA), fibronectin, IL-1β, collagen I, collagen IV (Abcam), Arkadia (Thermo Fisher), Smad7 (R&D Systems), phospho-NF-κB/p65, NF-κB/p65, and β-actin (Santa Cruz Biotechnology, CA). The membranes were incubated with the primary antibody overnight at 4 °C, and then stained with the LI-COR IRDye 800-labeled secondary antibodies (Rockland Immunochemicals, PA). The fluorescence intensity was detected by Odyssey Infrared Imaging System (Li-COR Biosciences, NE) and quantitated with the ImageJ program (NIH). The ratio for the examined protein was normalized against β-actin or total proteins as stated in the studies and is represented as mean ± SEM.

### ELISA

Kidney tissue was homogenized and the supernatant was collected for analysis by ELISA analysis. Level of TGF-β1 in plasma and renal tissues, including the active form, the latency-associated protein (LAP), and total TGF-β1, were quantitatively analyzed by commercial ELISA kits (R&D System Inc., Minneapolis, MN) as previously described 11.

### Statistical analyses

Data are presented as means ± standard error of the mean (SEM). Western blotting, immunostaining, real-time PCR were analyzed by one-way or two-way analysis of variance (ANOVA) followed by a multiple comparison test. UAE data were analyzed by repeated ANOVA. A value of *P* < 0.05 was considered statistically significant.

### Data and Resource Availability

The data sets generated in the study are available from the corresponding author upon reasonable request.

## Results

### Mice overexpressing latent TGF-β1 are protected against DKD

**Figure 1:**
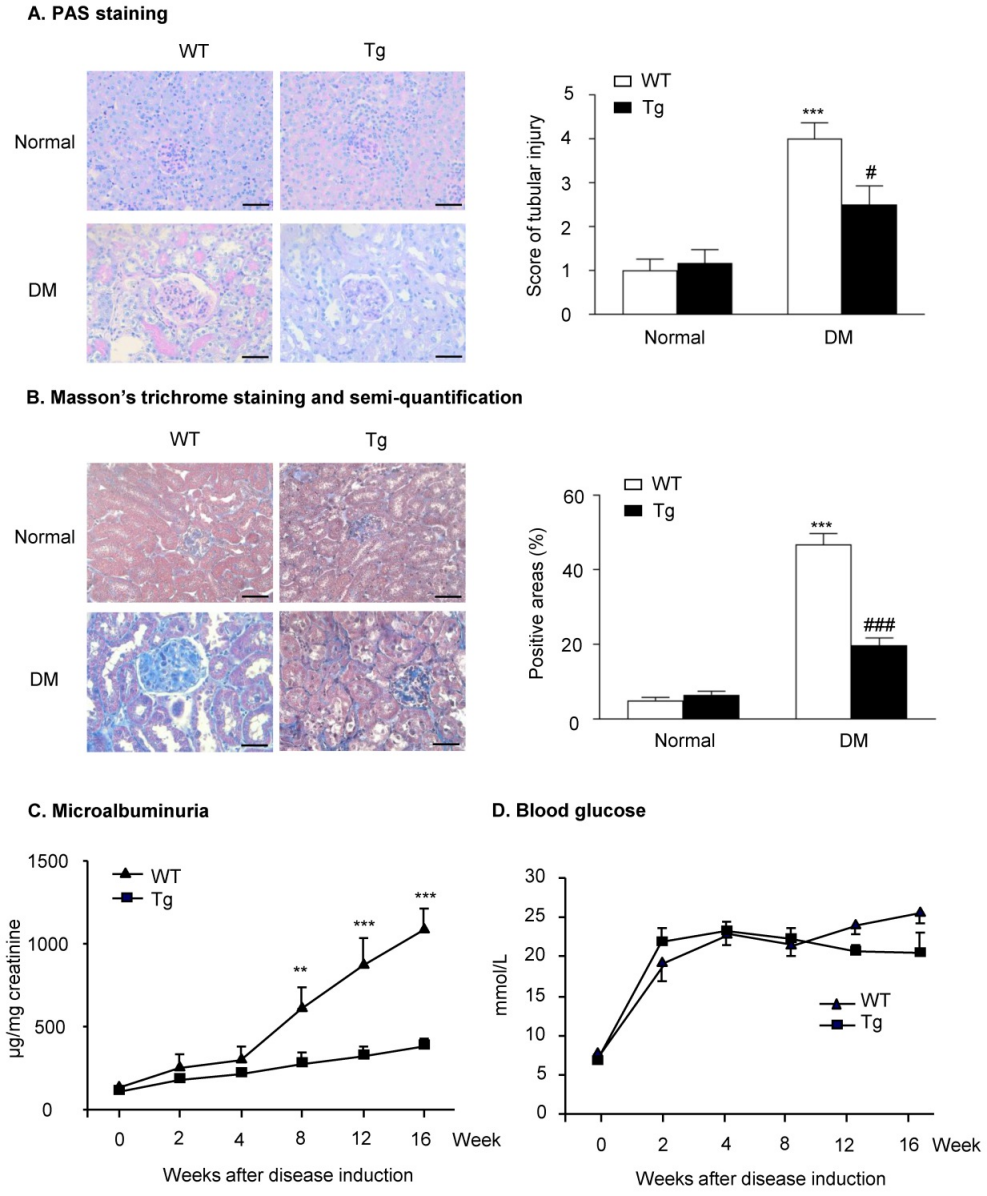
** Latent TGF-β1 attenuates kidney function in streptozotocin-induced type 1 diabetic mice.** Mice were euthanized 16 weeks after diabetes induction, and renal tissues were collected. **(A)** Periodic acid-Schiff (PAS) staining and score. Mesangial matrix expansion in glomeruli and the thickening of the glomerular basement membrane were observed. **(B)** Masson's trichrome staining and semi-quantification. Extracellular matrix deposition is shown in blue. **(C)** Microalbuminuria, and **(D)** Blood glucose levels in diabetic mice over 16 weeks. DM, diabetes mellitus. WT, latent TGF-β1 wild-type mice. Tg, latent TGF-β1 transgenic mice. Data represent the means ± SEM for groups of six animals. Scale bar: 50 µm. **P < 0.01, ***P < 0.001 versus normal mice; ^#^P < 0.05, ^###^P < 0.001 versus WT DM mice.

Histopathologically, the analysis of PAS staining and Masson's trichrome staining indicated that mice overexpressing latent TGF-β1 were protected against the development of mesangial matrix expansion and thickening of the glomerular basement membrane as seen in the diabetic kidneys of WT mice (**Figure 1A&B**). Similarly, the development of urinary microalbumin in the diabetic WT mice was also significantly inhibited in Tg mice with overexpressing latent TGF-β1 (**Figure 1C**). However, overexpression of latent TGF-β1 did not alter the levels of blood glucose (**Figure 1D**), indicating that latent TGF-β1 may protect against DKD locally without influencing the blood glucose. The levels of plasma latent TGF-β1 in both normal Tg and diabetic Tg mice were significantly higher compared to the WT mice, but the levels of active TGF-β1 had no significant difference between Tg and WT mice (**Supplementary Figure S1A&B**). In the kidneys, the levels of latent TGF-β1 in normal and diabetic mice are higher than the WT mice, while the level of active TGF-β1 was higher in diabetic WT mice compared to the diabetic Tg mice (**Supplementary Figure S1C&D**). The finding consistently indicated that latent TGF-β1 may protect against DKD locally.

### Overexpression of latent TGF-β1 attenuates renal fibrosis and inflammation in diabetic kidneys by suppressing the TGF-β/Smad and NF-κB signaling

**Figure 2:**
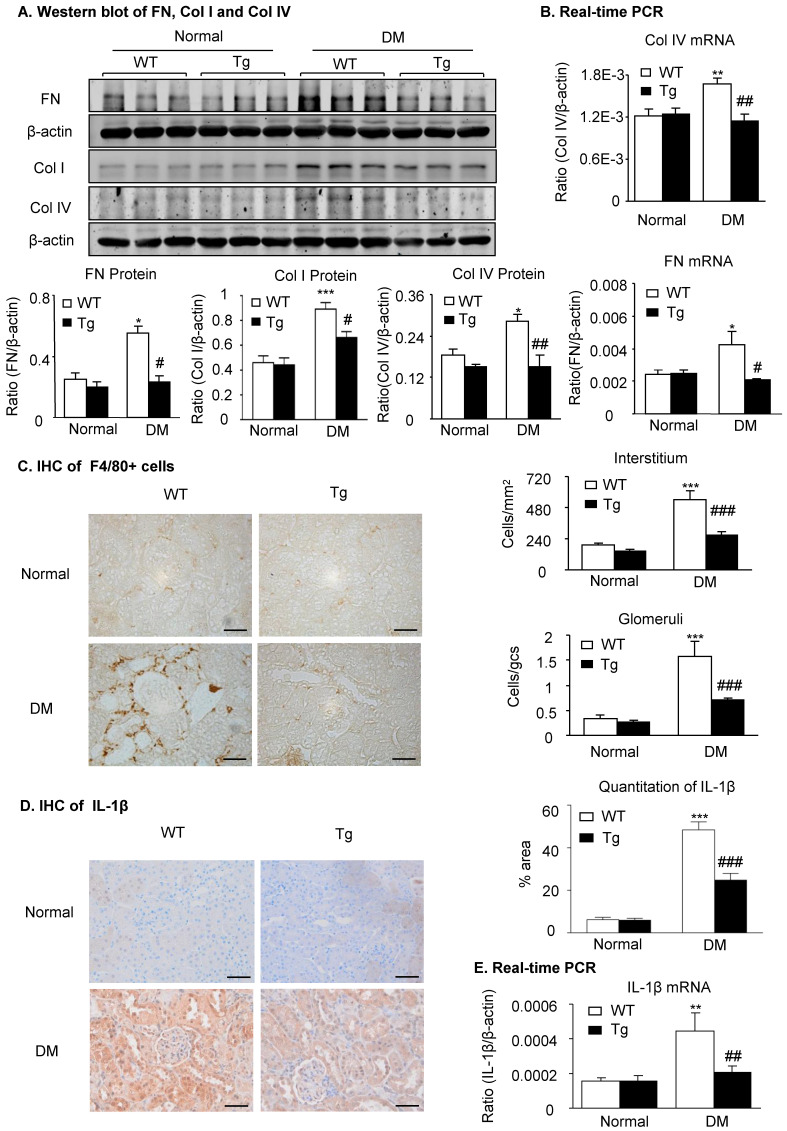
** Latent TGF-β1 attenuates renal fibrosis and inflammation in streptozotocin-induced type 1 diabetic mice.** Mice were euthanized 16 weeks after diabetes induction, and renal tissues were collected. **(A)** Protein and **(B)** mRNA expression of fibronectin (FN), collagen I (Col I) and collagen IV (Col IV) in normal and diabetic mice. **(C)** Immunohistochemistry of F4/80-positive cells showing macrophage infiltration in glomeruli and tubulointerstitium. **(D)** Immunohistochemistry and **(E)** mRNA expression of the inflammatory cytokine, interleukin-1β (IL-1β). DM, diabetes mellitus. WT, latent TGF-β1 wild-type mice. Tg, latent TGF-β1 transgenic mice. Data represent the means ± SEM for groups of six animals. Scale bar: 50 µm. *P < 0.05, **P < 0.01, ***P < 0.001 versus normal mice; ^#^P < 0.05, ^##^P < 0.01, ^###^P < 0.001 versus WT DM mice.

Because renal fibrosis and inflammation are two major pathological features determining the progression of DKD and are mediated by TGF-β/Smad3 and NF-κB signaling 2,14,17, we next examined whether overexpression of latent TGF-β1 inhibits renal fibrosis and inflammation by suppressing the TGF-β/Smad and NF-κB signaling. As shown in **Figure 2** and **Supplementary Figure S2**, immunohistochemistry, western blot, and real-time PCR detected that renal fibrosis such as increased expression of fibronectin, collagen I and collagen IV, the three major fibrosis markers in diabetic kidneys, was increased in WT mice but attenuated in latent TGF-β1 Tg mice. Similarly, renal inflammation including F4/80+ macrophages and expression of pro-inflammatory cytokines IL-1β and TNF-α was also increased in the diabetic kidneys of WT mice but blunted in the diabetic kidneys of Tg mice (**Figure 2C-E & Supplementary Figure S2D&E**).

**Figure 3:**
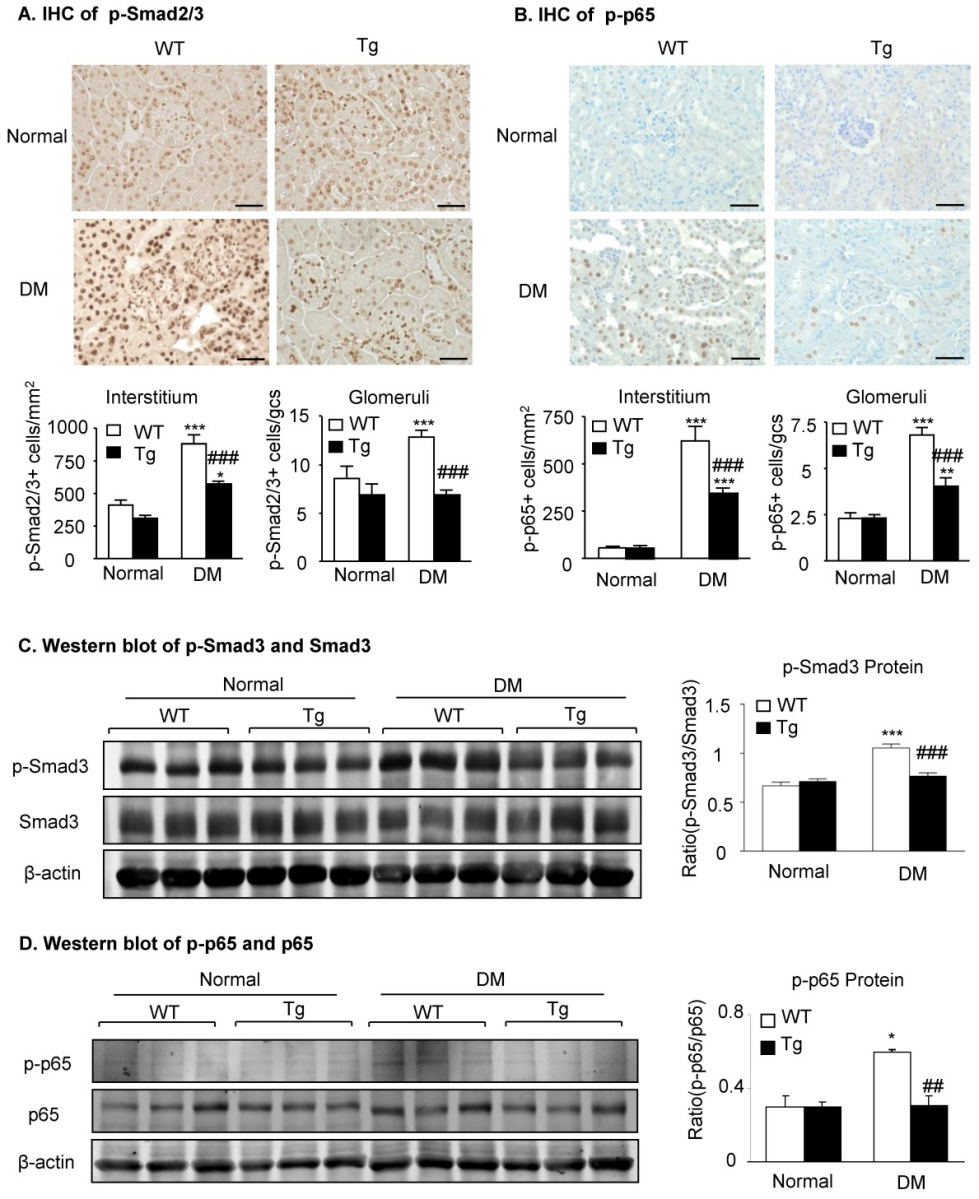
** Latent TGF-β1 attenuates diabetic kidney disease by suppressing TGF-β/Smad and NF-κB signaling.** Mice were euthanized 16 weeks after diabetes induction, and renal tissues were collected. **(A)** Smad2/3 nuclear translocation in different groups. **(B)** Phosphorylation of NF-κB p65 in glomeruli and tubulointerstitium. **(C)** Western blot analysis showing the phosphorylation of Smad3 in diabetic mice. **(D)** Phosphorylation of NF-κB p65. DM, diabetes mellitus. WT, latent TGF-β1 wild-type mice. Tg, latent TGF-β1 transgenic mice. Data represent the means ± SEM for groups of six animals. Scale bar: 50 µm. *P < 0.05, **P < 0.01, ***P < 0.001 versus normal mice; ^#^P < 0.05, ^##^P < 0.01, ^###^P < 0.001 versus WT DM mice.

Mechanistically, immunohistochemistry and western blot analysis detected that compared to diabetic WT mice, activation of both TGF-β/Smad3 and NF-κB/p65 signaling as evidenced by increased phosphorylation of Smad3 and NF-κB/p65 and their nuclear translocation in the diabetic kidneys was suppressed in the diabetic kidneys of Tg mice (**Figure 3**), demonstrating that latent TGF-β1 may protect against DKD by suppressing TGF-β/Smad-mediated renal fibrosis and NF-κB/p65-driven renal inflammation.

### Latent TGF-β1-induced suppression of Arkadia-mediated-Smad7 ubiquitin proteasomal degradation is a mechanism through which latent TGF-β1 Tg mice are protected against DKD *in vivo* and *in vitro*

**Figure 4:**
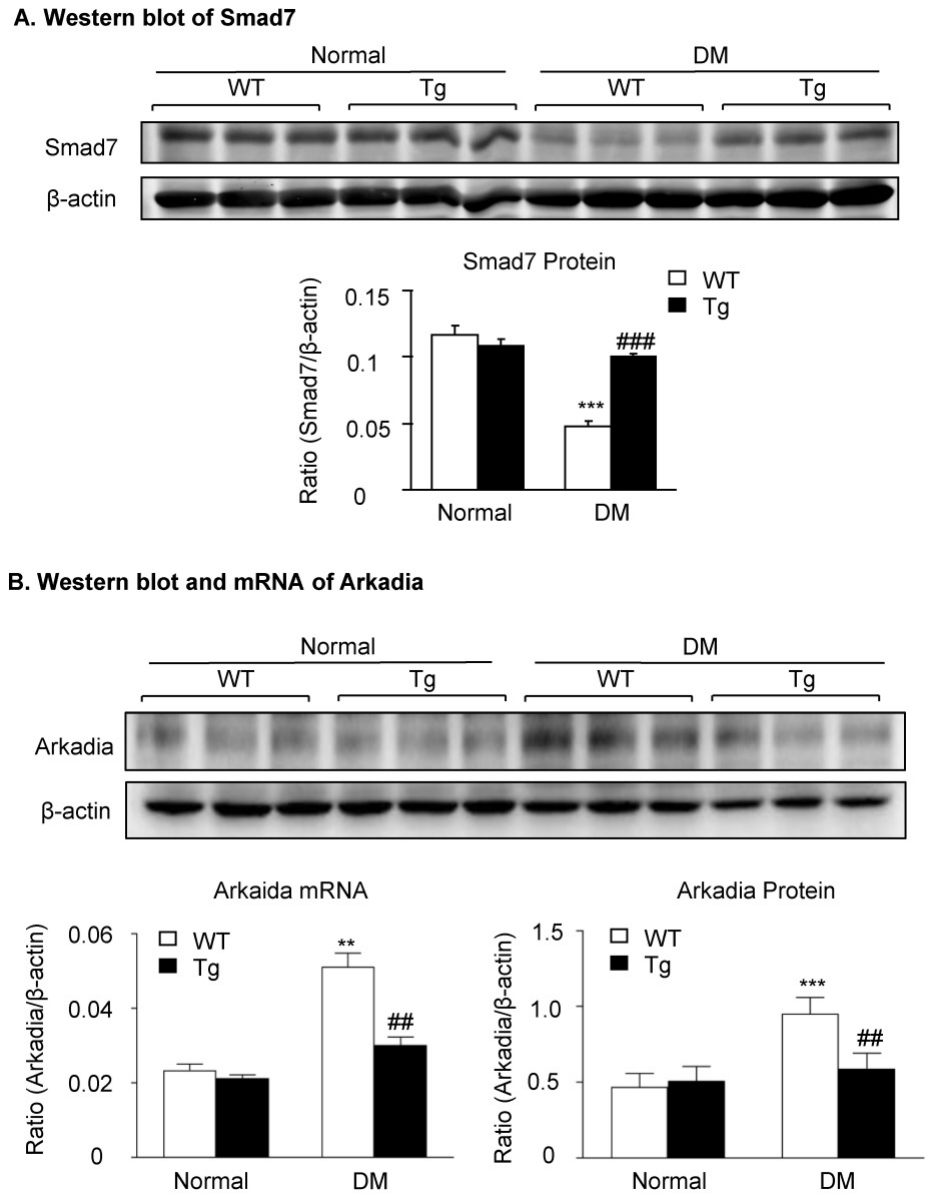
** Latent TGF-β1 suppresses TGF-β/Smad and NF-κB signaling, which is associated with the inhibition of Arkadia and restoration of Smad7 activity *in vivo*.** Mice were euthanized 16 weeks after diabetes induction, and renal tissues were collected. **(A)** Expression of Smad7 protein. **(B)** Expression of Arkadia protein and mRNA. DM, diabetes mellitus. WT, latent TGF-β1 wild-type mice. Tg, latent TGF-β1 transgenic mice. Data represent the means ± SEM for groups of six animals. *P < 0.05, **P < 0.01, ***P < 0.001 versus normal mice; ^#^P < 0.05, ^##^P < 0.01, ^###^P < 0.001 versus WT DM mice.

We have previously shown that renal Smad7 is essential for protection against DKD 14, and Smad7 ubiquitin-dependent proteasome degradation is a critical mechanism for renal fibrosis and inflammation in UUO 18. We thus examined whether inhibition of Smad7 ubiquitin-proteasome degradation may be a mechanism through which latent TGF-β1 Tg mice are protected against DKD *in vivo* and *in vitro*. As shown in **Figure 4**, western blot analysis detected that renal Smad7 was lost in the diabetic kidney of WT mice but was markedly increased in Tg mice (**Figure 4A**), which was inversely associated with an increase in the Arkadia, a ubiquitin E3-ligase targeting Smad7 for degradation (**Figure 4B**).

**Figure 5:**
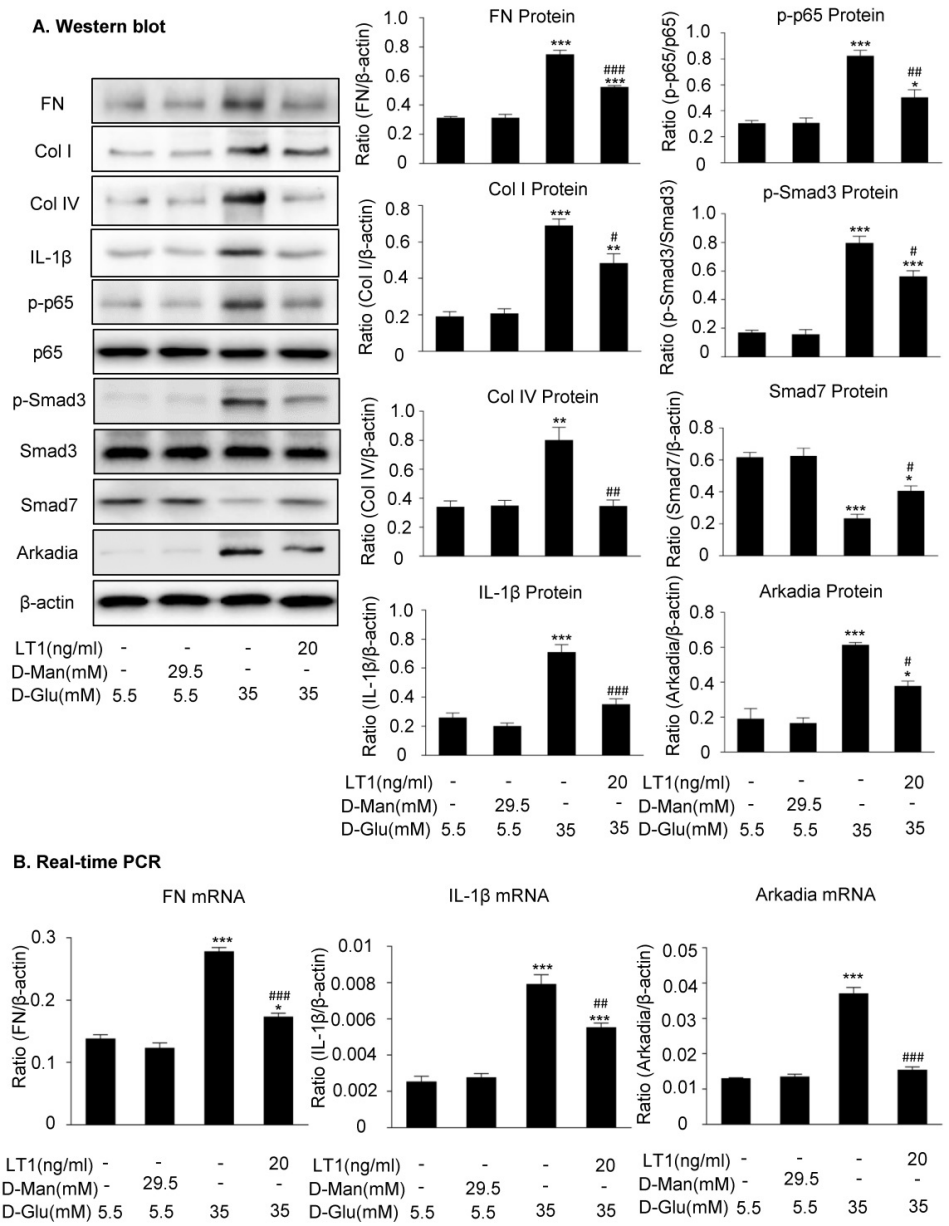
** Latent TGF-β1 suppresses TGF-β/Smad and NF-κB signaling, which is associated with the inhibition of Arkadia and restoration of Smad7 activity *in vitro*. (A)** Latent TGF-β1 reduced fibronectin, Col I, Col IV, IL-1β and Arkadia, suppressed the phosphorylation of Smad3 and NF-κB p65, and increased Smad7 expression in mouse mesangial cells (MCs) treated with high glucose. **(B)** Suppression of the expression of fibronectin, IL-1β, and Arkadia by latent TGF-β1. D-Man, D-mannitol (osmolality control). D-Glu, D-glucose. LT1, recombinant latent TGF-β1 protein (20 ng/ml). Data represent the means ± SEM from 3-4 independent experiments. *P < 0.05, **P < 0.01, ***P < 0.001 versus control; ^#^P < 0.05, ^##^P < 0.01, ^###^P < 0.001 versus 35 mM high D-glucose treatment.

**Figure 6:**
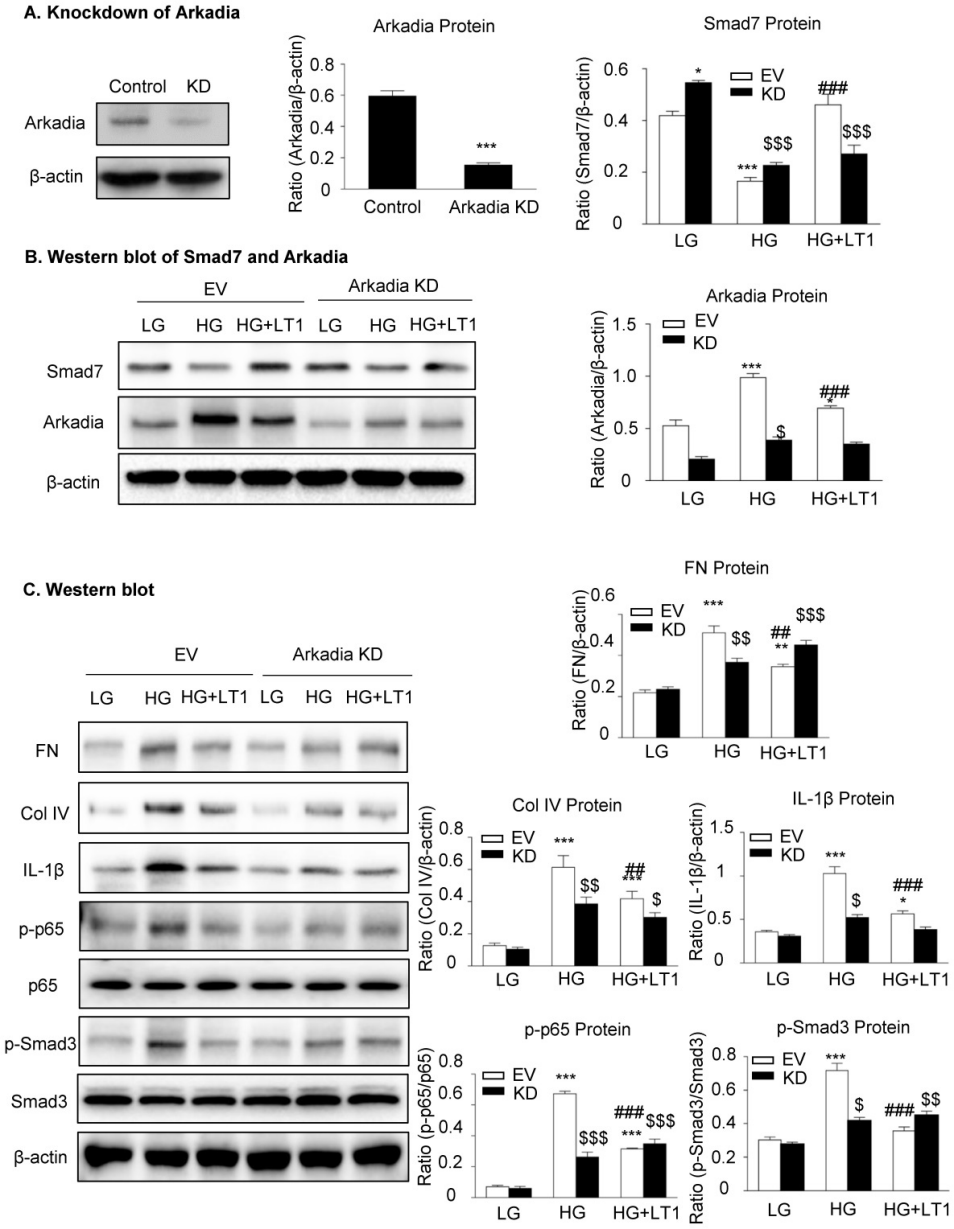
** The knockdown of Arkadia increases Smad7 and abolishes the effect of recombinant latent TGF-β1 protein on high glucose induced fibrosis and inflammation in cells. (A)** Knockdown of Arkadia in mesangial cells. **(B)** The level of Smad7 increased in mouse mesangial cells with Arkadia knockdown. **(C)** The knockdown of Arkadia in high glucose treated cells abolished the effect of latent TGF-β1. LG, low glucose (5.5 mM D-glucose). HG, high glucose (35 mM D-glucose). LT1, recombinant latent TGF-β1 protein (20 ng/ml). EV, Empty vector. KD, Knockdown. Data represent the means ± SEM from 3-4 independent experiments. *P < 0.05, **P < 0.01, ***P < 0.001 versus EV-LG control; ^#^P < 0.05, ^##^P < 0.01, ^###^P < 0.001 versus EV-HG treatment. ^$^P < 0.05, ^$$^ P < 0.01, ^$$$^ P < 0.001 versus KD-LG control.

**Figure 7:**
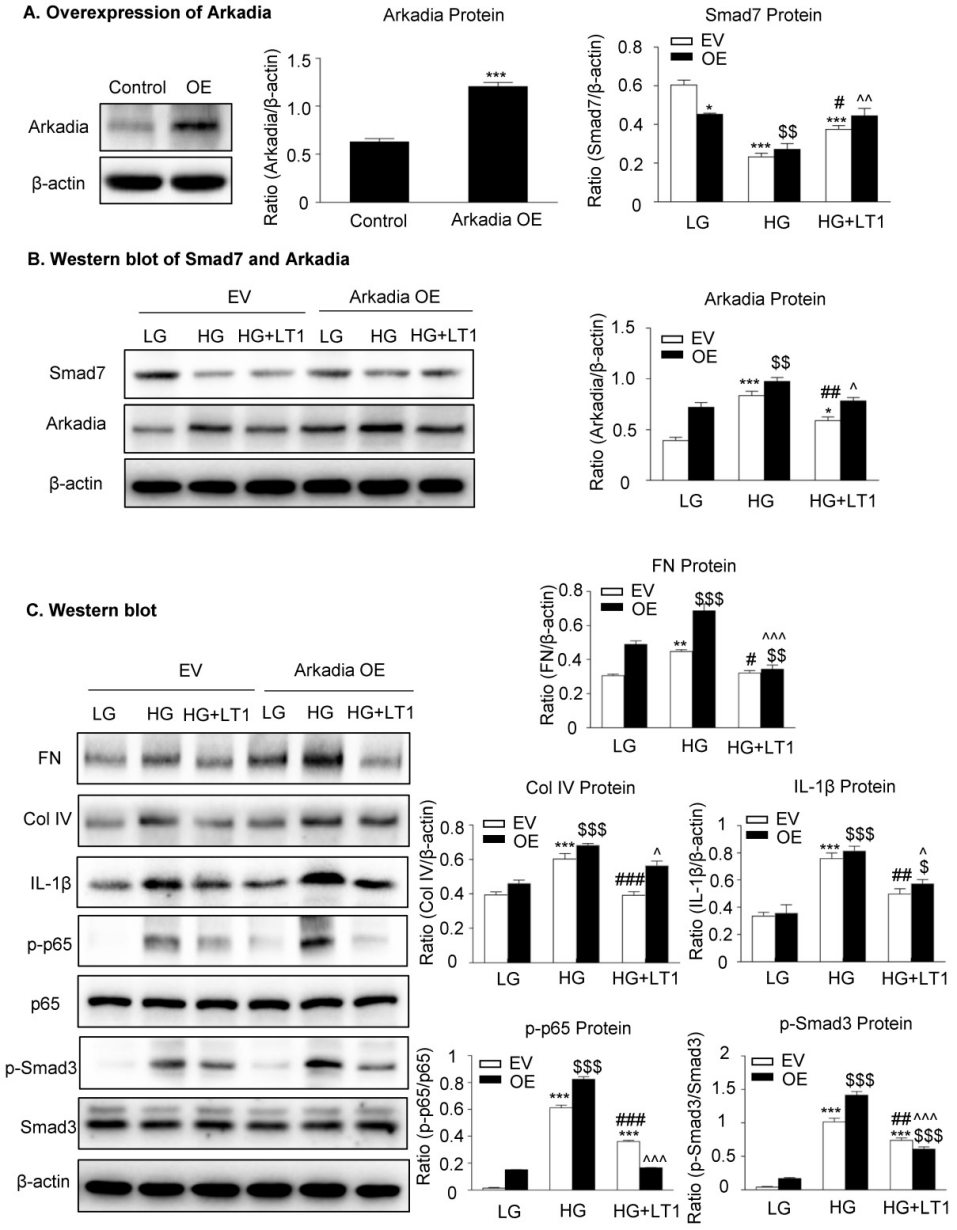
** The overexpression of Arkadia induces the loss of Smad7 and promotes the effect of recombinant latent TGF-β1 protein on high glucose-induced fibrosis and inflammation in cells, which inhibits Arkadia-mediated Smad7 degradation. (A)** Overexpression of Arkadia in mesangial cells. **(B)** The level of Smad7 decreased in mouse mesangial cells overexpressing Arkadia. **(C)** The addition of latent TGF-β1 to cells overexpressing Arkadia increased Smad7, which inhibited the fibrosis marker (fibronectin and Col IV) and inflammation marker (IL-1β) as well as inhibited the phosphorylation of Smad3 and NF-κB p65. LG, low glucose (5.5 mM D-glucose). HG, high glucose (35 mM D-glucose). LT1, recombinant latent TGF-β1 protein (20 ng/ml). EV, Empty vector. OE, Overexpression. Data represent the means ± SEM from 3-4 independent experiments. *P < 0.05, **P < 0.01, ***P < 0.001 versus EV-LG control; ^#^P < 0.05, ^##^P < 0.01, ^###^P < 0.001 versus EV-HG treatment. ^$^P < 0.05, ^$$^P < 0.01, ^$$$^ P < 0.001 versus OE-LG control. ^^^P < 0.05, ^^^^P < 0.01, ^^^P < 0.001 versus OE-HG treatment.

We then examined an essential role for Arkadia in mediating Smad7 degradation and the protective mechanisms of latent TGF-β1 under the high glucose conditions *in vitro*. As shown in **Figure 5 and Supplementary Figure S3,** the addition of latent TGF-β1 inhibited high glucose-induced expression of Arkadia but increased Smad7, resulting in inactivation of Smad3 and NF-κB/p65 signaling and reduction of fibronectin, collagen I, collagen IV and IL-1β expression. To determine the role of Arkadia in counter-regulating latent TGF-β1-mediated protective effects on diabetic renal injury by targeting Smad7, mesangial cells with Arkadia knockdown or overexpression were generated (**Figure 6A&7A**) and the regulatory role of Arkadia in Smad7 expression and activation of Smad3 and NF-κB signaling under high glucose and latent TGF-β1 conditions was investigated. As shown in **Figure 6B&C**, in mesangial cells with empty vector control, high glucose-induced loss of Smad7, phosphorylation of Smad3 and NF-κB/p65 signaling, and activation of pro-fibrotic and pro-inflammatory pathways were reversed by the addition of latent TGF-β1. The knockdown of Arkadia not only blocked high glucose-induced loss of Smad7, phosphorylation of both Smad3 and NF-κB/p65, and activation of pro-fibrotic and pro-inflammatory pathways, but also abolished the protective effect of latent TGF-β1 on these pathological changes. Conversely, overexpression of Arkadia significantly induced a loss of Smad7 in mesangial cells under the high glucose conditions, resulting in a marked activation of Smad3 and NF-κB/p65 signaling and upregulation of fibrosis markers (fibronectin, collagen IV) and pro-inflammatory cytokine IL-1β expression. Interestingly, the addition of latent TGF-β1 blocked Arkadia-mediated loss of Smad7 under high glucose conditions and therefore suppressed Smad3-mediated fibrosis (fibronectin) and NF-κB/p65-dependent IL-1β expression (**Figure 7B&C**).

### The renoprotective effect of latent TGF-β1 is probably through latency-associated peptide (LAP)

Latency-associated peptide noncovalently associates with mature TGF-β and forms latent TGF-β1 complex. To further explore the mechanism of latent TGF-β1-mediated protective effects on diabetic kidney disease, we used recombinant human LAP protein in our in-vitro study. As shown in **Supplementary Figure S4**, high glucose induced phosphorylation of Smad3 and NF-κB/p65 signaling in mouse mesangial cells, accompanied by loss of Smad7. Interestingly, the addition of LAP inhibited high glucose-induced Arkadia expression but increased Smad7, resulting in inactivation of Smad3 and NF-κB/p65 signaling, which was consistent with the latent TGF-β1 study, suggesting that the renoprotective effect of latent TGF-β1 is probably from the latency-associated peptide.

## Discussion

The present study demonstrated that latent TGF-β1 was renoprotective in DKD as mice overexpressing human latent TGF-β1 were protected against diabetic kidney disease by inhibiting TGF-β/Smad3-mediated fibrosis and NF-κB/p65-driven inflammation. This unexpected finding was further confirmed *in vitro* in which the addition of latent TGF-β1 was also capable of blocking high glucose-induced TGF-β/Smad3-mediated fibrosis and NF-κB/p65-driven inflammation in mesangial cells. Mechanistically, we uncovered that latent TGF-β1 protected against DKD by blocking E3-ligase Arkadia-mediated Smad7 ubiquitin proteasomal degradation pathway.

It is well established that TGF-β1 is profibrogenic in kidney fibrosis 15. In the canonic TGF-β pathway, once activated, TGF-β1 binds to its receptor II to activate the receptor I and the downstream regulatory Smad proteins (Smad2 and Smad3), facilitated by Co-Smad (Smad4), to translocate into the nucleus to initiate the target gene transcription and cause renal fibrosis, which is negatively regulated by Smad7 19,20. In the present study, we found that latent TGF-β1, unlike the active form of TGF-β1, plays a renoprotective role in DKD. Thus, the latent and active TGF-β1 exhibits an opposite role in DKD 21. We have previously reported that most of secreted TGF-β1 in the circulation is a latent form of TGF-β1 10,11. This may account for the failure of recent clinical trials by using the humanized monoclonal neutralizing antibodies against TGF-β1 (LY2382770) or all three isoforms of TGF-β (Fresolimumab) for treatment of patients with diabetic nephropathy 5 or FSGS 22,23. It is likely that the use of these neutralizing monoclonal antibodies may also block the renoprotective effects of latent TGF-β1. Results from this and the clinical trial studies suggest that treatment against renal fibrosis in patients with DKD should specifically target the downstream TGF-β signaling molecules rather than block the general effect of TGF-β1.

It is now clear that Smad7 is an integrated inhibitor not only for inhibiting TGF-β/Smad3-mediated renal fibrosis by competing with the R-Smad binding to the TβRI 24 but also for suppressing IκBα ng NF-κB-driven renal inflammation by inducing, an NF-κB inhibitor 25,26. We previously showed that renal Smad7 is lost in streptozotocin (STZ)-induced diabetic kidneys, which is associated with enhanced renal fibrosis and inflammation by activating the TGF-β/Smad3 and NF-κB signaling pathways, whereas the overexpression of Smad7 in diabetic kidneys attenuates TGF-β/Smad3-mediated renal fibrosis and NF-κB-driven renal inflammation 14. In addition, the loss of Smad7 expression also largely promotes TGF-β1-induced renal fibrosis and Smurf2/NF-κB-driven inflammation in mouse models of obstructive nephropathy and angiotensin II-induced hypertensive nephropathy 27-30. Arkadia, also known as ring finger 111 (Rnf111), is also an E3 ubiquitin ligase that promotes Smad7 degradation via Axin, a scaffold protein involved in TGF-β1 signaling 31. It has been reported that Arkadia can induce Smad7 degradation and thus enhance the renal tubular epithelial-to-mesenchymal transition and renal fibrosis in a rat unilateral ureteral obstruction model 32,33. In the present study, we found that Arkadia was significantly increased in the diabetic kidneys, which was associated with a loss of renal Smad7. In contrast, mice overexpressing latent TGF-β1 significantly increased Smad7. Exhibited high levels of renal Smad7 were associated with inhibition of renal Arkadia, suggesting that inactivation of the Arkadia-Smad7 ubiquitin proteasomal degradation pathway may be a key downstream mechanism through which latent TGF-β1 transgenic mice were protected against TGF-β/Smad3-mediated renal fibrosis and NF-κB-driven renal inflammation as previously reported 24-30. Further studies *in vitro* confirmed this notion as knockdown of Arkadia inhibited but overexpression of Arkadia promoted the renoprotective effects of latent TGF-β1 on TGF-β/Smad3-mediated fibrosis and NF-κB-dependent inflammation. Moreover, it was confirmed that the peptide of latent TGF-β1, LAP, inhibited Arkadia under high glucose conditions. Therefore, latent TGF-β1 has anti-fibrotic and anti-inflammatory effects in diabetic kidney injuries by suppressing Arkadia-mediated Smad7 degradation. LAP, the major peptide of latent TGF-β1, represents a potential target for the development of therapeutic approaches to treat DKD.

## Conclusion

In conclusion, latent TGF-β1 is renoprotective in STZ-induced type 1 diabetes. Inhibition of Arkadia-mediated renal Smad7 ubiquitin proteasomal degradation may be a mechanism through which latent TGF-β1 attenuates TGF-β/Smad3-mediated fibrosis and NF-κB p65-driven inflammation.

## Supplementary Material

Supplementary figures and tables.

## Abbreviations

LT1: Latent TGF-β1
LAP: Latency associated peptide
MC: Mesangial cell
NF-κB: Nuclear factor-κB
STZ: Streptozotocin
TGF-β: Transforming growth factor-β
WT: Wild-type
LG: Low glucose
HG: High glucose
