# Supplementary material for: Latent TGF-β1 protects against diabetic kidney disease via Arkadia/Smad7 signaling
Source: Int J Biol Sci. 2021 Aug 19;17(13):3583–94. doi: 10.7150/ijbs.61647 (PMC8416717; doi:10.7150/ijbs.61647)
Supplement: Supplementary file 1 — Supplementary figures and tables. [file ijbsv17p3583s1.pdf]

Supplementary Figures

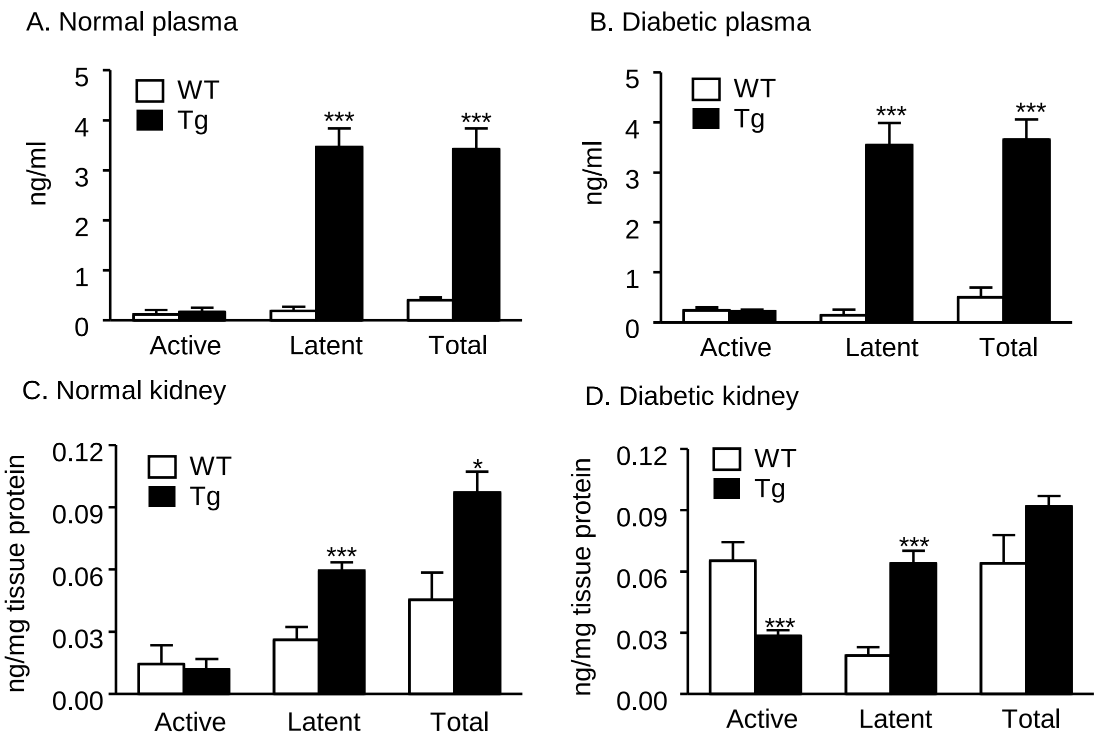

Figure S1. Levels of active, latent, and total TGF-β1 in plasma and kidney mice.

(A) Plasma levels in normal mice. **(B)** Plasma levels in diabetic mice. **(C)** Kidney levels in normal mice. **(D)** Kidney levels in diabetic mice. WT, latent TGF-β1 wild-type mice. Tg, latent TGF-β1 transgenic mice. Data represent the means ± SEM for groups of six animals. *$P < 0.05$, ***$P < 0.001$ Tg versus WT.

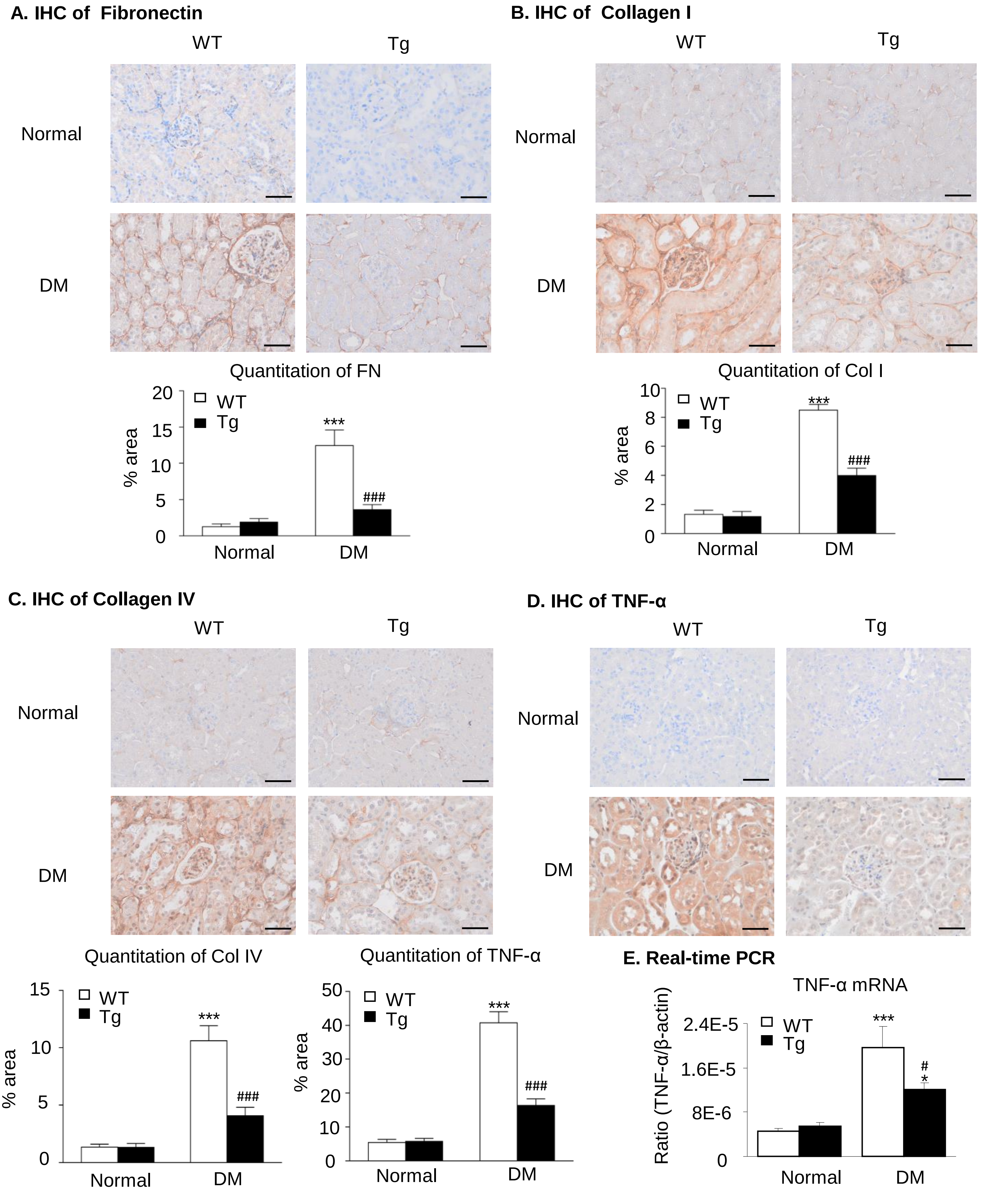

Figure S2. Latent TGF-β1 suppressed fibrosis and inflammation in diabetic kidneys.

Mice were euthanized 16 weeks after STZ injection, and renal tissues were collected. **(A)** Immunohistochemistry of Fibronectin. **(B)** Immunohistochemistry of Collagen I. **(C)** Immunohistochemistry of Collagen IV. **(D)** Immunohistochemistry of TNF-α. **(E)** Real-time PCR of inflammation index (TNF-α). DM, diabetes mellitus. WT, latent TGF-β1 wild-type mice. Tg, latent TGF-β1 transgenic mice. Data represent the means ± SEM for groups of six animals. Scale bar: 50 µm. *P < 0.05, **P < 0.01, ***P < 0.001 versus normal; $^{\#}$P < 0.05, $^{\#\#}$P < 0.01, $^{\#\#\#}$P < 0.001 versus WT-DM mice.

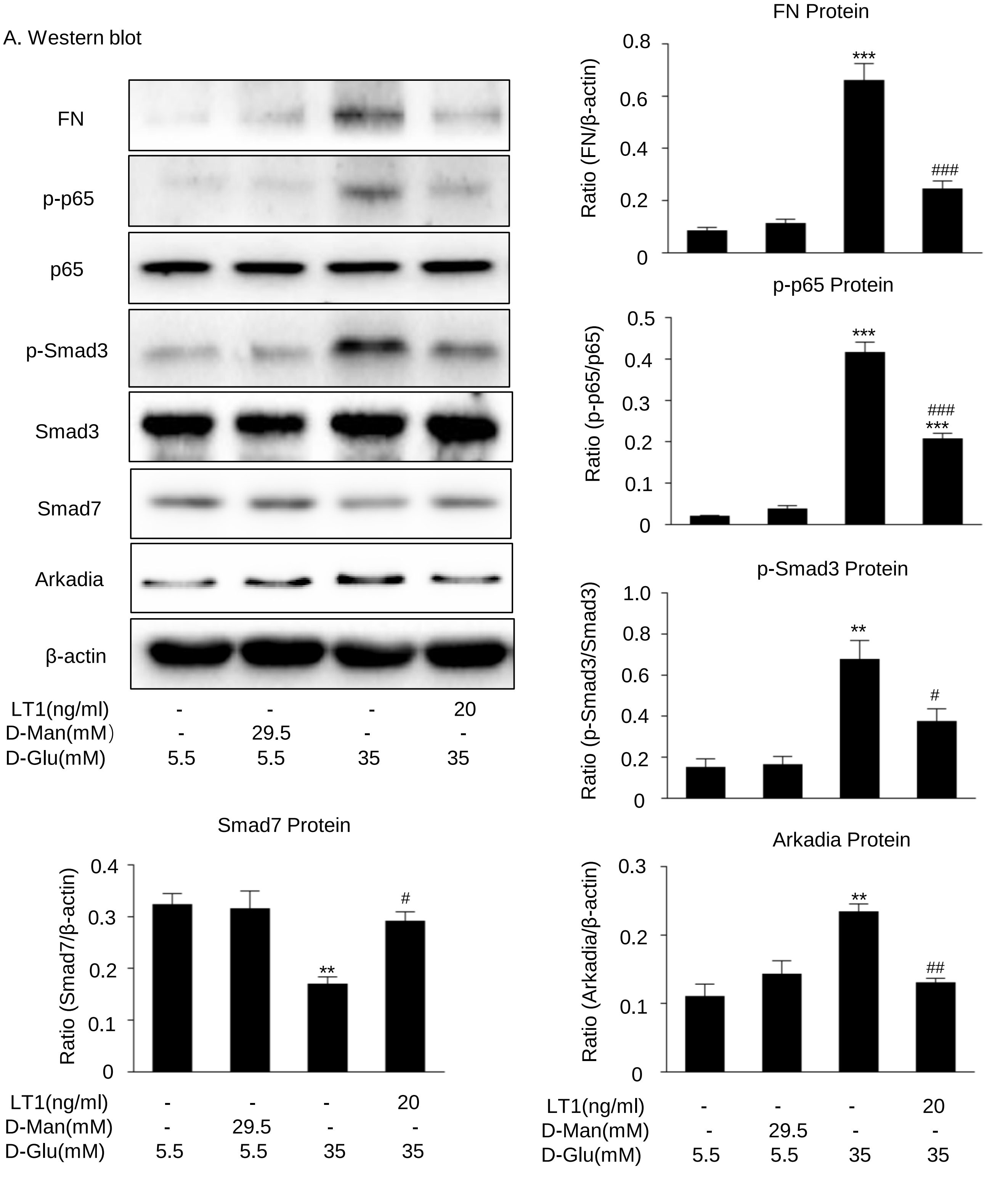

Figure S3 Latent TGF-β1 suppresses TGF-β/Smad3 and NF-κB signaling, which is associated with the inhibition of Arkadia and restoration of Smad7 activity in mTECs.

(A) Latent TGF-β1 reduced fibronectin, Col I, Col IV, IL-1β and Arkadia, suppressed the phosphorylation of Smad3 and NF-κB p65, and increased Smad7 expression in mouse tubular epithelial cells (mTECs) treated with high glucose. D-Man, D-mannitol (osmolality control). D-Glu, D-glucose. LT1, recombinant latent TGF-β1 protein (20 ng/ml). EV, Empty vector. KD, Knockdown. Data represent the means ± SEM from 3–4 independent experiments. *P < 0.05, **P < 0.01, ***P < 0.001 versus control; $^{\#}$P < 0.05, $^{\#\#}$P < 0.01, $^{\#\#\#}$P < 0.001 versus 35 mM high D-glucose treatment.

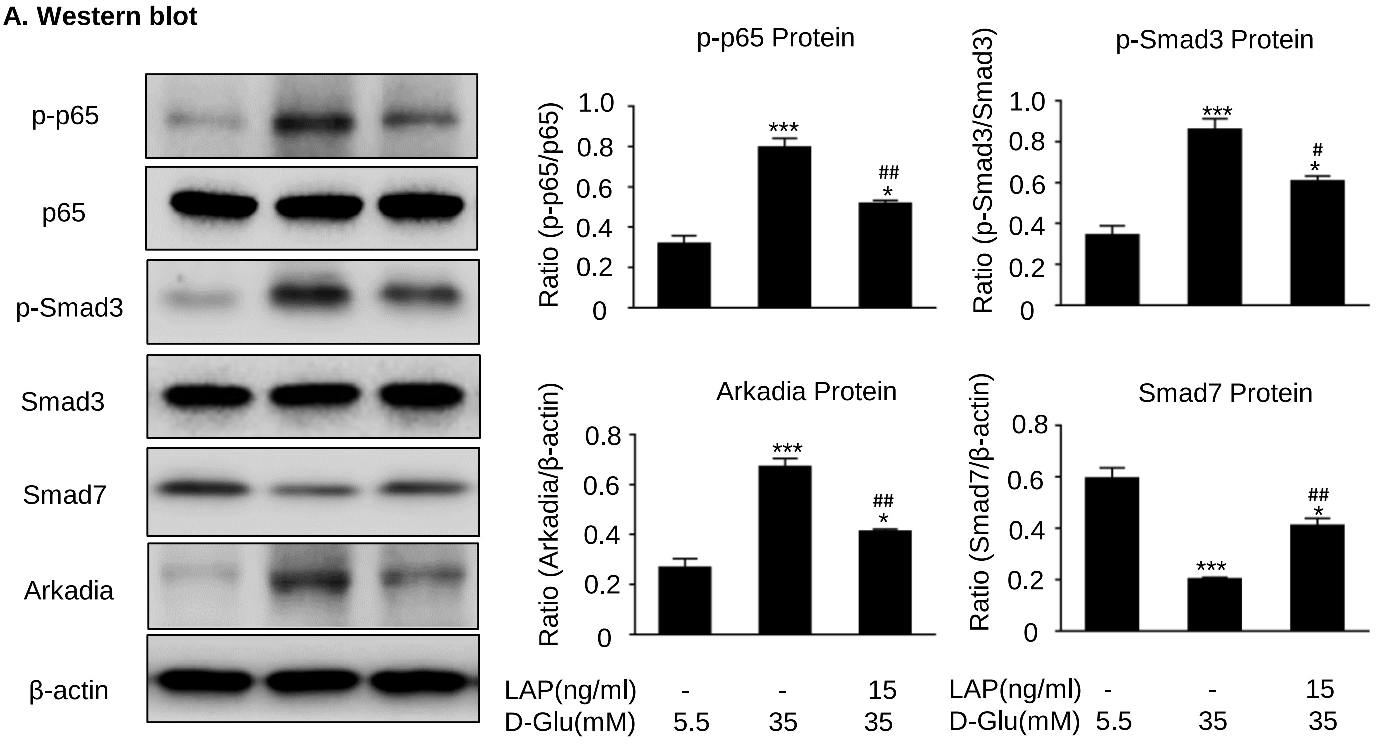

Figure S4 The renoprotective effect of latent TGF-β1 is probably through latency associated peptide (LAP).

(A) Western blot and quantitative analysis. LAP reduced the expression of Arkadia, suppressed the phosphorylation of Smad3 and NF-κB/p65, and increased Smad7 expression in mouse mesangial cells (MCs) treated with high glucose. D-Man, D-mannitol (osmolality control). D-Glu, D-glucose. LAP, recombinant latency associated peptide (15 ng/ml). Data represents the mean ± SEM for at least three independent experiments. *P < 0.05, **P < 0.01, ***P < 0.001 versus control; $^{\#}$P < 0.05, $^{\#\#}$P < 0.01, $^{\#\#\#}$P < 0.001 versus 35 mM high D-glucose treatment.

Supplementary Table 1. Primer sequences for qRT-PCR

	Gene	Primer sequence (5'-3')
Mouse	Col-IV	F: CTCAGGTCTCTGCTCAGAGCC
		R: CTGCGCTCCTCGTGGAGCAGAAG
Mouse	Fibronectin	F: TACCAAGGTCAATCCACACCCC
		R: CAGATGGCAAAAGAAAGCAGAGG
Mouse	TNF-α	F: CATGAGCACAGAAAGCATGATCCG
		R: AAGCAGGAATGAGAAGAGGCTGAG
Mouse	IL-1β	F: CTTCAGGCAGGCAGTATCACTCAT
		R: TCTAATGGGAACGTCACACACCAG
Mouse	Arkadia	F: CGACTTCATCACCTCCAGTTAG
		R: GCTCCATCCAATCCTGAAGAA
